# Early detection of infants with neurodevelopmental concerns indicative of cerebral palsy in a lower middle‐income country (India)

**DOI:** 10.1111/dmcn.16351

**Published:** 2025-06-15

**Authors:** Katherine A. Benfer, Asis K. Ghosh, Sayak Chowdhury, Golam Moula, Sandip Samanta, Pradip Maiti, Anjan Bhattacharya, Naila Zaman Khan, Koa Whittingham, Robert S. Ware, Cathy Morgan, Sasaka Bandaranayake, Iona Novak, Roslyn N. Boyd

**Affiliations:** ^1^ Queensland Cerebral Palsy and Rehabilitation Research Centre, Child Health Research Centre The University of Queensland Brisbane QLD Australia; ^2^ Indian Institute of Cerebral Palsy Kolkata India; ^3^ Institute of Post‐Graduate Medical Education and Research and Seth Sukhlal Karnani Memorial Hospital Kolkata India; ^4^ Asha Bhavan Centre Uluberia India; ^5^ Dr B C Roy Post Graduate Institute of Paediatric Sciences Kolkata India; ^6^ Child Development Centre Apollo Gleneagles Hospital Kolkata India; ^7^ Bangladesh Protibhondi Foundation Dhaka Bangladesh; ^8^ Menzies Health Institute Queensland Griffith University Brisbane QLD Australia; ^9^ Cerebral Palsy Alliance Research Institute, Specialty of Child & Adolescent Health, Sydney Medical School, Faculty of Medicine & Health The University of Sydney Sydney NSW Australia; ^10^ Queensland Paediatric Rehabilitation Service Queensland Children's Hospital Brisbane QLD Australia; ^11^ Faculty of Medicine & Health The University of Sydney Sydney NSW Australia

## Abstract

**Aim:**

To determine reproducibility and diagnostic accuracy of screening tools for neuromotor concerns indicative of cerebral palsy (CP) at 18 months corrected age by using the General Movements Assessment (GMA) and/or Hammersmith Infant Neurological Examination (HINE) in West Bengal, India.

**Method:**

This prospective substudy tested psychometrics of screening tools nested within an overarching randomized control trial. A total of 785 infants with birth/infant‐detectable risk factors, aged 12 to 40 weeks corrected age (*n* = 422 male, mean corrected age 22.6 weeks, SD = 10.2), were recruited. Infants were screened for ‘high‐risk CP’ using the GMA (absent/abnormal fidgety, 12–17 weeks corrected age) and/or HINE (3 months < 56, 6 months < 59, 9 months < 62, 18–40 weeks corrected age). ‘Neuromotor concerns indicative of CP’ were classified at 18 months corrected age by a physician from a videoed neurological examination and semi‐structured movement protocol. We analysed the results (1) using Gwet's AC1 and (2) for sensitivity and specificity.

**Results:**

Interrater reproducibility was strong (Gwet's AC1 = 0.89, *p* < 0.001). A total of 165 out of 749 assessments were screened as ‘high‐risk CP’ (22.0%; 95% confidence interval 19.2–25.1). The screening programme (GMA/HINE) was 80.1% accurate (GMA [only] sensitivity = 87.8%, specificity = 44.4%; HINE [only] sensitivity = 94.0%, specificity = 60.0%).

**Interpretation:**

The GMA and/or HINE are reliable and accurate tools for screening high‐risk populations in India, and may be useful in other low‐ and middle‐income countries to identify infants with neuromotor concerns indicative of CP who could be triaged to early intervention.

## Graphical abstract

In this prospective sub‐study testing the predictive validity of General Movements Assessment (GMA) and/or Hammersmith Infant Neurological Examination (HINE), 785 infants with birth/infant‐detectable risk factors, aged 12 to 40 weeks corrected age were recruited. GMA and HINE were reliable and accurate tools for screening high‐risk populations in India, and may be useful in other low‐ and middle‐income countries to identify infants with neuromotor concerns indicative of cerebral palsy who could be triaged to early intervention .

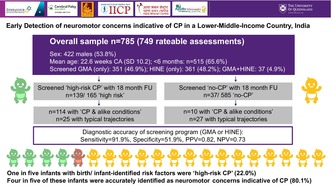

**This original article is commented on by Damiano on pages** 1519‐1520 **of this issue.**

**Abbreviations**
CPG: clinical practice guideline
GMA: General Movements Assessment
HIC: high‐income country
HINE: Hammersmith Infant Neurological Examination
LEAP‐CP: Learning through Everyday Activities with Parents for infants at risk of Cerebral Palsy
LMIC: low‐ and middle‐income country
RCT: randomized controlled trial

**What this paper adds**

- Twenty‐two per cent of infants with risk factors screened as having ‘high‐risk of cerebral palsy’ (CP) in West Bengal, India.
- There was high screening uptake (94%), with 66% screened at younger than 6 months and 95.4% rateable assessments.
- Interrater reproducibility was strong for General Movements Assessment (GMA) and/or Hammersmith Infant Neurological Examination (HINE).
- GMA/HINE was 80% accurate for predicting neuromotor concerns indicative of CP.

Cerebral palsy (CP) is among the most common and costly of childhood physical disabilities. Early identification of infants at ‘high‐risk of CP’ in the first year of life is recommended, on the basis of high‐quality evidence in the international clinical practice guideline (CPG) for CP.^1^, ^2^ This ensures infants can be fast‐tracked to receive targeted early interventions during a period of rapid neural development and plasticity.^3^ A meta‐analysis of implementation studies adhering to the CPG in high‐income countries (HICs) has shown a high effect size for lowering the age at diagnosis to 7.5 months.^4^ Less is known, however, about implementation of the CPG in low‐ and middle‐income countries (LMICs).

Approximately 80% of the global burden of CP is in LMICs, owing to larger populations and higher rates of CP.^5^, ^6^ This is further compounded by more complex clinical profiles, and an array of social determinants and service access barriers (economic, geographical, attitudinal, informational), leading to later diagnosis and subsequent access to intervention, and poorer functional outcomes.^7^, ^8^, ^9^, ^10^ Health systems in LMICs are often under significant pressure to service large and complex caseloads, with limitations to physical resources and workforce capacity (time, medical education, centralized specialist services).^11^, ^12^ There is a significant need for low‐cost, accessible, and scalable early disability screening models feasible for LMICs in order to implement the CPG recommendations.^11^

To make an early clinical diagnosis of ‘high risk of CP’, the CPG recommends that the essential criterion is met of ‘motor dysfunction’ using tools with strong predictive validity, in combination with clinical history indicating risk of CP or abnormal neuroimaging.^1^ Both the General Movements Assessment (GMA) and the Hammersmith Infant Neurological Examination (HINE) are among the most predictive, and are objective, quick and easy to administer, and offer a low‐cost means for regular surveillance once GMA certification is obtained.^13^, ^14^ Access to GMA Trust certification (3‐day course) is increasingly available in LMICs, with certified raters in more than 40 countries (C Einspieler, personal communication 2021). The HINE was designed to be appropriate for cross‐cultural settings, with training videos freely available online.^15^ The reproducibility and diagnostic accuracy of the GMA and/or HINE in LMICs is not well‐researched.^16^ Published research in Africa, Asia, Eastern Europe, and South America report generally strong but varied diagnostic accuracy (GMA fidgety predicted later developmental outcome: median sensitivity = 80%, specificity = 96%;^11^, ^17^, ^18^, ^19^, ^20^, ^21^, ^22^, ^23^, ^24^, ^25^ HINE scores <67 at 12–42 weeks predicted CP diagnosis at 2 years: sensitivity = 83%, specificity = 88%).^22^ The most predictive HINE scores for infants aged 5 to 6 months corrected age in Sri Lanka were calculated to be not more than 58.5, which yielded sensitivity of 89.5% and specificity of 79.7%.^25^ This nested substudy implemented CPG‐recommended screening tools for LMIC (GMA and/or HINE) and aimed to determine (1) reproducibility and (2) diagnostic accuracy to detect neuromotor concerns indicative of CP in a high‐risk population with birth/infant‐detectable risks in a lower middle‐income country (West Bengal, India). The primary hypothesis was that the GMA and/or HINE would have equivalent prediction of neuromotor concerns indicative of CP (sensitivity/specificity) compared with HIC literature.

## METHOD

### Study design

This prospective diagnostic study (March 2017–August 2018) tested screening tools in a geographical sample of infants with birth/infant‐detectable risks residing in urban and rural communities in West Bengal, India. It was a nested substudy of the overarching randomized controlled trial (RCT) of LEAP‐CP early intervention (Learning through Everyday Activities with Parents for infants at ‘risk of Cerebral Palsy’).^26^ Ethics approval was obtained through the Children's Health Queensland Hospital and Health Service Human Research Ethics Committee (HREC/16/QRCH/214), The University of Queensland Medical Research Ethics Committee (2016001073), Apollo Gleneagles Hospital Kolkata Institutional Ethics Committee (IEC/2016/12/35), and Dr. B C Roy Postgraduate Institute Institutional Ethics Committee (BCH/ME/PR/54). All families gave written informed consent to participate. The study had clinical trial registration ANZCTR 12616000653460.

#### Participants

Infants aged 12 to 40 weeks corrected age with parent‐reported or physician‐recorded birth/infant‐detectable risk factors (Appendix S1) residing in study communities (Appendix S2) were eligible. Infants with known or suspected congenital/chromosomal abnormalities likely to affect neurodevelopment, or with neurodegenerative conditions, or infants considered medically fragile (including frequent/long‐term hospitalization) were excluded.

Infants were screened with the GMA (GMA only, if screened ‘predicted no‐CP’), HINE (HINE only, if entering study at ≥18 weeks corrected age), or GMA plus HINE (if screened ‘high‐risk of CP’) until achieving RCT target recruitment (*n* = 142).^26^ All infants identified as ‘high‐risk of CP’ during screening were invited to participate in the RCT. Final outcome data at 18 months corrected age from all infants screening ‘high‐risk of CP’ who entered the RCT were used to determine the diagnostic accuracy for identifying neuromotor concerns indicative of CP. In addition, infants screened as ‘predicted no‐CP’ on the GMA/HINE (excluded from RCT), aged 18 months corrected age from July 2018 to March 2019 were invited to return for follow‐up. The ‘predicted no‐CP’ sample was selected using computer‐generated random number sequences until achieving the target sample size. Sample size was calculated on the basis of estimates of GMA and HINE sensitivity/specificity (sensitivity = 0.95, specificity = 0.96;^27^ sensitivity = 0.90, specificity = 0.89;^28^ respectively) to demonstrate HIC‐equivalent diagnostic accuracy to ±10% precision (95% confidence interval [CI]). A total of 19 GMA infants at ‘high‐risk of CP’, 15 ‘predicted no‐CP’, and 35 HINE infants at ‘high‐risk of CP’, 38 ‘predicted no‐CP’, were needed.

#### Procedures

Infants with birth/infant‐detectable risk factors were identified for screening in the community through field visits by community disability workers (*n* = 11) using multiple identification strategies, including expansion of existing services (routine immunization clinics, primary health centres, community antenatal/primary care), conducting community activities/education alongside recruitment, and household screening. Infants in the hospital were identified by discharge record review and screened at special care nursery follow‐up clinics.

A single GMA video was collected when the infant was ‘fidgety age’ (12–17 weeks corrected age) by community disability workers who were employed for the trial (many were already employed by partner organizations) and given a weekly honorarium. Community disability workers received basic training on calculating corrected age and the essential criteria for filming GMA across three practical sessions, and did not start data collection until competency was achieved. In the hospital, GMA videos were collected by a paediatric physiotherapist (GM Trust certified). Infants screened as ‘high‐risk of CP’ on the GMA were also assessed using the HINE, with no further screening provided to infants screened as ‘predicted no‐CP’. Infants who were identified as at least 18 weeks corrected age were screened only with the HINE. The HINE was administered in person by one of four trained Indian clinicians (who were trained online with the ‘HINE Train the Trainer’ course, L M Hataaja, University of Helsinki; and who co‐administered HINE items for the first three infants with the first author [KAB] to ensure fidelity), with all assessments digitally recorded. Two trained therapists (advanced GMA certified, including reliability testing) scored all GMA and HINE videos (one Indian [AKG], one Australian independently), with a third rating when scores lacked consensus (GMA) or were ± 2 points of cut‐scores (HINE) (a clinician with >10 years' experience in the tools). Dual‐ratings were used for calculating reproducibility. At 18 months corrected age, infants were reassessed on HINE, and a semi‐standardized movement observation videoed by the local research assistant (SC) to elicit skills necessary for diagnosis and to classify with the Gross Motor Function Classification System (GMFCS).

#### Early detection measures

The GMA observes the quality of spontaneous infant movement patterns which form a reliable indicator of brain dysfunction at both the writhing (preterm to 6–9 weeks postterm age) and fidgety (12–17 weeks corrected age) periods. Infants with absent/abnormal fidgety movements on GMA were considered as having ‘high‐risk of CP’ as per the CPG.^29^ The psychometrics of the GMA are well documented in HICs, with sensitivity = 95% to 100%, specificity = 96% to 98%,^27^ and interrater reproducibility kappa = 0.91 to 0.92, average agreement 90%.^14^

The HINE is a standardized quantifiable neurological examination for infants aged 2 to 24 months corrected age consisting of 26 items (cranial nerves, posture, movement, tone, reflexes). Infants scoring below published HINE CP cut‐points^30^ were categorized ‘high‐risk of CP’ (<56.3 months sensitivity = 96%, specificity = 85%; <59.6 months sensitivity 90%, specificity 89%; <62.9 months sensitivity = 90%, specificity = 91%).^28^ The HINE has excellent intra‐ and interrater reliability for global scores (intraclass correlation coefficient = 0.97) when administered to 12‐month‐old high‐risk infants in India.^31^

#### Baseline measures

A physician medical checklist was collected from the caregiver/medical record (as available, retrospective data) by the research assistant (SC). This included sociodemographic, birth (preterm status, complications), and developmental history (medications, comorbidities on the 10 Question Screen).^32^

#### Outcome measures

Outcomes at 18 months corrected age were classified by an Australian paediatrician blinded to medical history from a videoed neurological examination and semi‐standardized movement observation, as was conducted in our previous work.^30^ The paediatrician completing the classifications was not trained in the HINE and did not refer to scores to confirm diagnosis. The videoed HINE was only used to systematically observe tonal and postural differences in the child's musculoskeletal system and movement. Consensus rating for ‘uncertain’ cases was completed independently by an experienced physiotherapist with more than 30 years' experience in CP classification (RNB).

Classification of neuromotor concerns indicative of CP was provided according to published guidelines,^33^, ^34^, ^35^ classified as ‘definite or probable’ (‘probable’ defined as the blinded clinical presentation most likely reflective of CP, but not representing a common motor pattern associated with CP diagnosis, including equinus gait, scissoring legs, crouch gait, upper limb pronation, thumb adduction, wrist flexion, fisting during hand grasp) or ‘not‐CP’ (blinded clinical presentation related to typical development/genetic aetiology).

The GMFCS (<2 years age band), a five‐level classification, was used to categorize children's gross motor function.^36^ Motor type (spasticity, dyskinesia, ataxia, hypotonia) and distribution (number of limbs) were classified according to Surveillance of CP in Europe guidelines.^35^ Differential identification of the primary motor type from video in mixed classifications (spasticity and dystonia) was not possible, therefore a ‘mixed’ classification was included.

### Statistical analysis

Demographics and the proportion of infants screened ‘high‐risk of CP’ were presented descriptively, with 95% CIs (Stata version 16; StataCorp, College Station, TX, USA). Interrater reproducibility was assessed using Gwet's AC1 (owing to an unbalanced sample) and percentage agreement (data dichotomized to ‘high‐risk of CP’/‘predicted no‐CP’), and intraclass correlation coefficients for HINE optimality scores. Diagnostic accuracy (sensitivity, specificity, positive predictive value, negative predictive value) were calculated for (1) GMA plus HINE, (2) GMA (only), (3) HINE (only), and (4) screening (GMA or HINE) to detect neuromotor concerns indicative of CP at 18 months corrected age. Significance was set at *p* < 0.05, with no adjustment for multiple testing.

## RESULTS

A total of 834 infants were referred, with 785 screened (807 assessments completed; *n* = 37 GMA plus HINE, *n* = 351 GMA only, *n* = 361 HINE only). Infant and family demographics are presented in Table 1, recruitment pathways and reasons not screened in Figure S1, and analysis of infants lost to follow‐up in Table S1 and Table S2 (no systematic differences based on sex, age, neurological severity, *p* > 0.05). The recruitment rate was high (94.1%), with 515 infants (65.6%) screened at younger than 6 months corrected age (mean age 22.6 weeks corrected age). A total of 749 assessments were rateable on the GMA or HINE (95.4%), with only 37 infants having GMA plus HINE (reasons for single screening tool: *n* = 314 infants screened on GMA were ‘predicted no‐CP’ [GMA only, as per protocol], *n* = 361 screened ≥18 weeks corrected age [HINE only, as per protocol], *n* = 37 missed datapoint).

**TABLE 1:** Baseline characteristics of sample of infants assessed in early detection of cerebral palsy in West Bengal, India (*n* = 785).

	*n* (%)
Sex, male	422 (53.8)
Corrected age in weeks at assessment, mean (SD)	22.6 (10.2)
**Study site**	
Child In Need Institute	223 (28.4)
Asha Bhavan Centre	266 (33.9)
BC Roy	296 (37.7)
*Birth risk factors* ^a^	
Total number of risk factors, mean (SD)	2.2 (1.2)
Infection in mother	10 (1.3)
Infection in infant	67 (8.5)
Preterm birth (born <37 weeks gestational age)	186 (23.7)
Moderate–late preterm (32–37 weeks)	126 (18.0)
Very preterm (28–32 weeks)	27 (3.9)
Extremely preterm (<28 weeks)	7 (1.0)
Preterm classification unknown	26 (3.3)
Weeks gestation, mean (SD)	38.3 (2.7)
Low birthweight (<2500 g)	228 (29.0)
Mean birthweight, kg, mean (SD)	1.8 (0.4)
HIE or birth asphyxia	222 (28.3)
Level	
HIE I	7 (3.2)
HIE II	53 (23.9)
HIE III	7 (3.2)
HIE unspecified	155 (69.8)
Seizures within 72 hours of birth	68 (8.7)
Neonatal jaundice	275 (35.0)
NICU or special care nursery admission	477 (60.8)
Days in NICU, mean (SD)	5.7 (5.6)
Head injury in infant	5 (0.6)
Other	188 (23.9)
*Infant identifiable risk factors*	
Altered tone/asymmetry^b^	8 (1.0)
Delayed motor milestones^b^	11 (1.4)
Other	6 (0.8)

Abbreviations: BC Roy, Dr. B C Roy Postgraduate Institute for Paediatric Sciences; HIE hypoxic–ischaemic encephalopathy; NICU, neonatal intensive care unit; SD standard deviation.

^a^ Multiple risk factors may be present in one infant.

^b^ These were only recorded if the primary reason for referral (i.e. no other birth risk factors were present). Missing data: sex *n* = 2, corrected age *n* = 18, gestational age *n* = 84, mean birthweight *n* = 68, HIE level *n* = 155, days in NICU *n* = 350.

### Proportion of infants screened ‘high‐risk of CP’

A total of 165 infants were identified as having ‘high‐risk of CP’ on either screening tool (22.0%; 95% CI 19.2–25.1), 74 on GMA (19.0%; 95% CI 15.4–23.2), and 91 on HINE (25.2%; 95% CI 21.0–30.0). The mean HINE score was 63.4 (SD = 16.5, range 6.5–78), with scores significantly lower at hospital and rural sites than the urban site (mean 59.7 [SD = 16.7], *p* < 0.001 [univariate linear regression]; 64.1 [SD = 16.1], *p* = 0.05; 68.4 [SD = 15.8] respectively).

### Interrater reproducibility of early detection tools

Interrater reproducibility between two raters (from a pool of four) of ‘high‐risk of CP’ classification was ‘almost perfect’ for overall screening (GMA or HINE; Gwet's AC1 = 0.89 [95% CI 0.86–0.92], 92.7% agreement, *p* < 0.001), for GMA only (Gwet's AC1 = 0.86 [95% CI 0.82–0.91], 90.8% agreement, *p* < 0.001), and HINE only (<CP cut‐point [see Method]: Gwet's AC1 = 0.92 [95% CI 0.88–0.96], 94.9% agreement, *p* < 0.001; score intraclass correlation coefficient = 0.85 [95% CI 0.76–0.93], *p* < 0.001).

### Diagnostic accuracy of early detection tools

A total of 176 infants had follow‐up at 18 months for diagnosis; 139 out of 165 infants were screened as ‘high‐risk of CP’ and an additional 37 as ‘predicted no‐CP’. Infants screened with the GMA plus HINE had 15 out of 37 in agreement (40.5%) with 12 out of 15 (80%) accurately predicting outcome of neuromotor concerns indicative of CP. Use of individual screening tools yielded diagnostic accuracy of GMA only (sensitivity = 87.8%, specificity = 44.4%, positive predictive value = 0.71, negative predictive value = 0.71) and HINE only (sensitivity = 94.0%, specificity = 60.0%, positive predictive value = 0.89, negative predictive value = 0.75) (Table 2). Almost three‐quarters of infants with neuromotor concerns indicative of CP (70.2%) were classified as non‐ambulatory CP (GMFCS levels IV–V), most (74.3%) presenting with bilateral spasticity four‐limb involvement (27.7%), dyskinesia (25.5%), or mixed motor type with bilateral spasticity and four‐limb involvement (21.3%) (Table 3).

**TABLE 2:** Diagnostic accuracy of international clinical practice guideline‐recommended screening tools for detecting infants with neuromotor concerns indicative of cerebral palsy in West Bengal, India.

	Total screened (*n*)	True positives (*n*)	True negatives (*n*)	False positives (*n*)	False negatives (*n*)	Sensitivity, % (95% CI)	Specificity, % (95% CI)	PPV^a^	NPV^a^
GMA (only) to detect neuromotor concerns	68	36	12	15	5	87.8 (73.8–95.9)	44.4 (25.5–64.7)	0.71	0.71
Asha Bhavan Centre	20	11	3	3	3	78.6 (49.2–95.3)	50.0 (11.8–88.2)	0.79	0.50
BC Roy	34	19	8	7	0	100.0 (82.4–100.0)	53.3 (26.6–78.7)	0.73	1.00
CINI	14	6	1	5	2	75.0 (34.9–96.8)	16.7 (0.4–64.1)	0.55	0.33
HINE (only) to detect neuromotor concerns	108	78	15	10	5	94.0 (86.5–98.0)	60.0 (38.7–78.9)	0.89	0.75
Asha Bhavan Centre	40	27	9	1	3	90.0 (73.5–97.9)	90.0 (55.5–99.8)	0.96	0.75
BC Roy	56	43	4	8	1	97.7 (88.0–99.9)	33.3 (9.9–65.1)	0.84	0.80
CINI	12	8	2	1	1	88.9 (51.8–99.7)	66.7 (9.4–99.2)	0.89	0.67
Screening (GMA or HINE) to detect neuromotor concerns	176	114	27	25	10	91.9 (85.7–96.1)	51.9 (37.6–66.0)	0.82	0.73
Asha Bhavan Centre	60	38	12	4	6	86.4 (72.7–94.8)	75.0 (47.6–92.7)	0.91	0.67
BC Roy	90	62	12	15	1	98.4 (91.5–100.0)	44.4 (25.5–64.7)	0.81	0.92
CINI	26	14	3	6	3	82.4 (56.6–96.2)	33.3 (7.5–70.1)	0.70	0.50

*Note*: HINE scores were available for 30 out of 45 of the children who were not diagnosed with neuromotor concerns indicative of cerebral palsy, with *n* = 8 scoring below the optimality score of 73.^13^

Abbreviations: BC Roy, Dr. B C Roy Postgraduate Institute for Paediatric Sciences; CI, confidence interval; CINI, Child In Need Institute; GMA, General Movements Assessment; HINE, Hammersmith Infant Neurological Examination; NPV, negative predictive value; PPV, positive predictive value.

^a^ PPV/NPV calculations based on ratio of cases in current sample. ‘Neuromotor concerns indicative of cerebral palsy’ included possible mild cerebral palsy (*n* = 11), microcephaly (*n* = 1), hydro−/macrocephaly (*n* = 6), motor delay (*n* = 7), hypotonia/ataxia (*n* = 1), suspected syndrome (*n* = 2), toe walker (*n* = 1).

**TABLE 3:** Motor severity and type of infants identified with neuromotor concerns indicative of cerebral palsy and infants ‘screened at high risk of cerebral palsy’ in West Bengal, India.

	Neuromotor concerns indicative of CP at 18 months corrected age (*n* = 94)	‘High risk of CP’ on GMA or HINE (*n* = 141)	‘High risk of CP’ on GMA (*n* = 53)	‘High risk of CP’ on HINE (*n* = 88)
GMFCS level				
I	1 (1.1)	39 (27.7)	26 (49.1)	13 (14.8)
II	17 (18.1)	24 (17.0)	6 (11.3)	18 (20.5)
III	10 (10.6)	11 (7.8)	2 (3.4)	9 (10.2)
IV	19 (20.2)	20 (14.2)	7 (13.2)	13 (14.8)
V	47 (50.0)	47 (33.3)	12 (22.6)	35 (39.8)
Primary motor type^a^				
Unilateral spasticity	4 (4.3)			
Bilateral spasticity (2 limbs)	2 (2.1)			
Bilateral spasticity (3+ limbs)	26 (27.7)			
Dyskinetic	24 (25.5)			
Mixed spasticity/dystonia	20 (21.3)			
Hypotonic	11 (11.7)			
Ataxic	7 (7.5)			
Comorbidities at baseline^a^				
Epilepsy	47 (50.0)			
Vision	48 (51.1)			
Hearing	39 (41.5)			
Cognitive	55 (58.5)			

*Note*: Data presented as *n* (%).

Abbreviations: CP, cerebral palsy; GMA, General Movements Assessment; GMFCS, Gross Motor Function Classification System; HINE, Hammersmith Infant Neurological Examination.

^a^ Primary motor type and comorbidities only classified for cases with movement patterns indicative of CP diagnosis.

## DISCUSSION

This was a prospective nested substudy of an RCT conducted in vulnerable low‐resource communities in West Bengal, India. The findings demonstrated that early screening for infants with birth/infant‐detectable risk factors using tools recommended in the CPG was reliable and accurate for identifying neuromotor concerns indicative of CP at 18 months. Our early detection‐RCT recruitment strategy in high‐risk populations achieved high recruitment rates (94.1%), high rates of scorable assessments (95.4%), high interrater agreement (92.7%), and high accuracy for predicting neuromotor concerns indicative of CP (80.1%).

The ability of GMA‐fidgety to accurately identify later diagnosis was strong (70.6%), despite only collecting a single GMA video, and its frequent use in isolation from the HINE (single tool only). The GMA (only) was highly sensitive when used according to our protocol, although the specificity was less than 50%. Literature reports are variable for diagnostic accuracy of GMA‐fidgety compared with developmental outcome at 12 to 24 months when used in LMICs, with sensitivity ranging from 28% to 100% (median = 80%) and specificity from 67% to 100% (median = 96%).^11^, ^17^, ^18^, ^19^, ^20^, ^21^, ^22^, ^23^, ^24^, ^25^ Numerous factors could influence this variability including infant risk factors, underlying population prevalence of CP, reliability of raters, as well as the type and timing of outcomes. In our sample, we found significant variability in the diagnostic accuracy of GMA even between study sites, particularly hospital versus community screening; with specificity lowest in the urban slum site. While mostly anecdotal, in the community sites there was greater reliance on parents to accurately provide the infant's birth history and corrected age, greater variability in recording environment and number of clinicians administering the screening, significantly lower proportion of infants with more predictive birth risk factors (20% hypoxic‐ischemic encephalopathy or birth asphyxia in the community GMA vs. 68% in the hospital, 5% seizures in the community GMA vs. 31% in the hospital), and lower rates of CP diagnosis.

The diagnostic accuracy of the HINE (only) for identifying neuromotor concerns indicative of CP was strong, and better than that of the GMA. Our reported accuracy was almost equivalent to data reported in HICs of 90%,^28^ as well as other LMIC data (sensitivity = 83%, specificity = 87% with cut‐score less than 67 to detect CP at 2 years; sensitivity = 89.5%, specificity = 79.7% with cut‐score less than 58.5 to detect CP at 2 years).^22^, ^25^ It is interesting to note that research on the Hammersmith Neonatal Neurological Examination (equivalent assessment for infants aged <2 months) has reported significantly lower scores when administered in Ghana compared with British norms, with only 5% of ‘not‐CP’ term‐born neonates in Ghana classified as neurologically optimal.^37^ This may, in part, provide a plausible explanation for false positives on the HINE in our sample compared with HICs. Future research to investigate modified cut‐points for prediction in particular LMICs is warranted.

Many early detection protocols are reporting better accuracy of prediction when multiple results are triangulated, particularly when combined with imaging.^25^, ^38^ Our findings reported equivalent prediction (80%) when using the GMA plus HINE (in agreement) compared with the screening programme using GMA or HINE. It is important to note that our a priori protocol was that an infant who screened as ‘predicted no‐CP’ on the GMA did not require further assessment (on the HINE); as such, only 20% of cases had both tools. As these numbers are low, the results should be interpreted cautiously. Furthermore, the combined GMA plus HINE results were only available for ‘predicted CP’ cases, and this would also skew the results and limit their interpretation.

Almost three‐quarters of infants with neuromotor concerns indicative of CP at 18 months were classified as non‐ambulatory (GMFCS levels IV–V; with bilateral spasticity four‐limb involvement, dyskinesia, or mixed spasticity‐dystonia). The rates of infants with unilateral spasticity and bilateral spasticity with two‐limb involvement which are typically associated with mild perinatal brain injury were low in our sample; however, these are reflective of the lower rates of infants born very/extremely preterm in our cohort.^39^ Approximately half of the infants had one or more comorbidities including epilepsy, visual impairment, hearing impairment, or cognitive impairment. There have been four population‐based studies in LMICs, which described the clinical profile of CP (Uganda in children aged 2–18 years; India <10 years; Turkey 2–16 years; Bangladesh <18 years).^6^, ^10^, ^40^, ^41^ Rates of bilateral spasticity with four‐limb involvement/dyskinesia in these reports ranged from 26% to 42% (compared with HIC = 30.6%); non‐ambulant CP 20% to 46% (HIC = 25.5%);^6^, ^10^ and comorbidities of epilepsy 21% to 23% (HIC = 30%), visual impairment 4% to 10% (HIC = 36.4%), hearing impairment 10% to 12% (HIC = 12.8%), and cognitive impairment 26% to 39% (49.0%).^6^, ^10^, ^42^ Our sample of children had significantly poorer functional abilities compared with these population‐based data. This may have been due to our recruitment strategy, with approximately half the sample recruited from a tertiary hospital, and/or lower accessing of services for infants with mild functional profiles, which skewed sampling towards infants with severe functional profiles. In addition, the neuromotor concerns indicative of CP were classified from video (subtle tone may have been under‐identified with possible misclassification of mild presentations as typical development), and the age of our cohort was young (which could have resulted in survival bias, with high rates of infant mortality, particularly in severe cases).

There were some key limitations to our research which may have affected the accuracy and generalizability of our findings. As this nested substudy was situated within a larger RCT, it was beyond the scope to follow‐up all infants for diagnosis. Infants with ‘predicted no‐CP’ who agreed to follow‐up (70% of invited infants) may have been more likely to attend owing to developmental concerns (although our analysis of infants with follow‐up vs. no follow‐up suggested no systematic bias). To minimize burden on local medical teams and ensure consistency across sites, outcomes were classified on the basis of videoed assessment. This may have limited identification of mild tone differences, reducing the reported accuracy of the tests. Infants in this cohort all had birth/infant‐identifiable risk factors; as such, rates may not be generalizable to a population estimate. Infants identified as ‘high‐risk of CP’ were included in the RCT (half receiving active early intervention). While accessing LEAP‐CP may have reduced the functional severity of infants, there is no evidence that accessing behavioural/allied‐health interventions reduces CP diagnosis. Finally, there were limitations to the quality of the screening data, including incomplete medical history for determining study exclusion, potential inaccuracy of parent‐reported corrected age, collection of only a single GMA video (and a limited number of infants having GMA plus HINE available to triangulate), and HINE scored from video. These factors probably reduced the predictive validity reported in this real‐world study in West Bengal, India.

## CONCLUSION

With high uptake of the screening and high rates of scorable assessments in more than 800 administrations, this study provides data on CPG‐recommended screening tools to reduce the age of identification of neuromotor concerns indicative of CP to younger than 6 months which may be feasible for other LMIC high‐risk populations. The local clinicians were able to implement the screening programme with fidelity, following access to globally recognized training from expert trainers, and support from experienced clinicians during the implementation (RNB, KM, KAB). Certification training and continuing support are necessary to ensure calibration for accuracy of ratings, reflected in the strong interrater reproducibility. By delivering the screening through local community members, it enabled a feasible, free, home‐based service. Upskilling a broader network of GMA administrators, for example nurses who conduct routine immunization and government antenatal workers, would enhance coverage and reduce geographical barriers. This strategy and/or the use of a phone app may also enable easier access to a second GMA video ideally triangulated with the HINE which could improve the accuracy of identification. A large gap in the screening completeness was due to loss of contact with families owing to change in contact details/migration. This may be mitigated by linking families with a community disability worker/health worker from the local community at the time of hospital discharge. Screening should always be provided with the support of a medical team to ensure appropriate referrals (for both child and family, including mental health services/support), and sensitive communication of diagnostic information. The strong predictive validity found in the present study should be tested in larger studies, and could be further strengthened through exploring the feasibility of implementing CPG Pathway B with GMA (two videos) plus HINE and/or a motor assessment. Further development and upscaling of accessible early intervention/family support models such as LEAP‐CP^43^ for LMICs is also critical if such screening programmes are to make a significant difference to the lives of infants with CP and their families in these settings.

## FUNDING INFORMATION

The LEAP‐CP study is funded by grants from the Cerebral Palsy Alliance Australia, QEII Diamond Jubilee Endeavour scholarship, Commonwealth Government Australia (KAB); Merchant Charitable Funding; Australian National Health and Medical Research Council (NHMRC) Early Career Grant (KAB) 1195602 and Investigator grant (RNB).

## CONFLICT OF INTEREST STATEMENT

The authors have stated that they had no interests that might be perceived as posing a conflict or bias.

## Supporting information


**Table S1:** Infants with ‘high‐risk of cerebral palsy’ (*n* = 165).

**Table S2:** Infants with ‘predicted no‐cerebral palsy’ (*n* = 620).

**Figure S1:** Recruitment pathways of the LEAP‐CP detection substudy.

**Appendix S1:** Birth‐ and infant‐detectable risk factors for screening.

**Appendix S2:** Geographical catchments of screening.

## Data Availability

De‐identified individual participant data that underlie the results reported in this article will be available following publication of primary RCT results to researchers submitting a methodologically sound proposal.
