# Supplementary material for: Early detection of infants with neurodevelopmental concerns indicative of cerebral palsy in a lower middle‐income country (India)
Source: Dev Med Child Neurol. 2025 Jun 15;67(12):1554–63. doi: 10.1111/dmcn.16351 (PMC12618952; doi:10.1111/dmcn.16351)
Supplement: Supplementary file 1 — Table S1: Infants with ‘high‐risk of cerebral palsy’ (n = 165). [file DMCN-67-1554-s004.docx]

**Table S1. Infants with ‘high-risk of cerebral palsy’ (n=165)**

|  | **Infants with FU**  **n=139** | **Infants without FU**  **n=26** | **P value** |
| --- | --- | --- | --- |
| Sex (proportion, 95% CI) | 56.2 (47.7, 64.3) | 50.0 (31.5, 68.5) | 0.55 |
| Age of screening weeks (mean, SD) | 25.9 (11.9) | 21.5 (10.8) | 0.09 |
| Neurological severity (mean, SD)# | 38.0 (15.4) | 40.5 (10.9) | 0.64 |

#HINE at baseline available for n=87
