# Supplementary material for: Early detection of infants with neurodevelopmental concerns indicative of cerebral palsy in a lower middle‐income country (India)
Source: Dev Med Child Neurol. 2025 Jun 15;67(12):1554–63. doi: 10.1111/dmcn.16351 (PMC12618952; doi:10.1111/dmcn.16351)
Supplement: Supplementary file 2 — Table S2: Infants with ‘predicted no‐cerebral palsy’ (n = 620). [file DMCN-67-1554-s003.docx]

**Table S2. Infants with ‘predicted no-cerebral palsy’ (n=620)**

|  | **Infants with FU**  **n=37** | **Infants without FU**  **n=583** | **P value** |
| --- | --- | --- | --- |
| Sex (proportion, 95% CI)* | 56.4 (40.2, 71.3) | 53.8 (49.6, 57.9) | 0.75 |
| Age of screening weeks (mean, SD) | 23.0 (8.3) | 22.2 (9.8) | 0.61 |
| Neurological severity (mean, SD)^ | 69.3 (5.5) | 71.3 (5.7) | 0.10 |

* Data available for n=543; ^ HINE at baseline available for n=284
