# Supplementary figures and images for: Early detection of infants with neurodevelopmental concerns indicative of cerebral palsy in a lower middle‐income country (India)
Source: Dev Med Child Neurol. 2025 Jun 15;67(12):1554–63. doi: 10.1111/dmcn.16351 (PMC12618952; doi:10.1111/dmcn.16351)

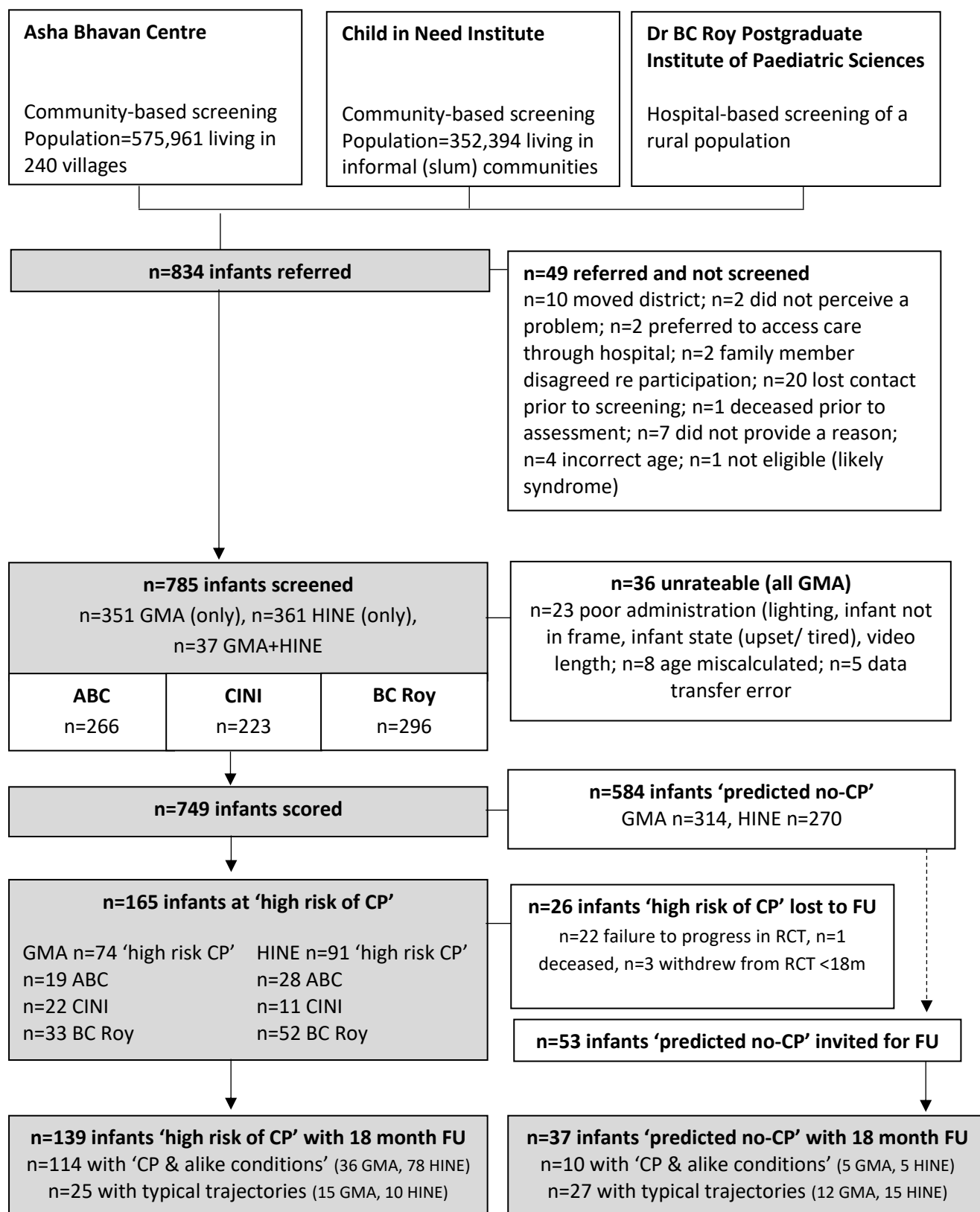

Supplement: Supplementary file 3 — Figure S1: Recruitment pathways of the LEAP‐CP detection substudy. [file DMCN-67-1554-s005.pdf]
