# Supplementary material for: Early detection of infants with neurodevelopmental concerns indicative of cerebral palsy in a lower middle‐income country (India)
Source: Dev Med Child Neurol. 2025 Jun 15;67(12):1554–63. doi: 10.1111/dmcn.16351 (PMC12618952; doi:10.1111/dmcn.16351)
Supplement: Supplementary file 4 — Appendix S1: Birth‐ and infant‐detectable risk factors for screening. [file DMCN-67-1554-s001.docx]

**Appendix S1. Birth- and infant-detectable risk factors for screening**

The risk factors were defined broadly to allow for parent report in a context with lower health literacy. Specific risk factors included:

1. Antenatal maternal infection at any time during the pregnancy
2. Low birth weight (LBW, <2·5kg)
3. Preterm delivery (<37 weeks gestational age, based on last menstrual period)
4. Hypoxic Ischaemic Encephalopathy (HIE)
5. Perinatal birth asphyxia (BA)
6. Neonatal jaundice requiring treatment (levels not defined)
7. Seizures within 72 hours after birth
8. Admission to Neonatal Intensive Care Unit or Special Care Nursery
9. Post-neonatal complications in infant (infection, head injury, near drowning)
10. Altered muscle tone/ delayed motor milestones for the infant (as reported by parents or allied health/ medical professionals).
