# Supplementary material for: Early detection of infants with neurodevelopmental concerns indicative of cerebral palsy in a lower middle‐income country (India)
Source: Dev Med Child Neurol. 2025 Jun 15;67(12):1554–63. doi: 10.1111/dmcn.16351 (PMC12618952; doi:10.1111/dmcn.16351)
Supplement: Supplementary file 5 — Appendix S2: Geographical catchments of screening. [file DMCN-67-1554-s002.docx]

**Appendix S2. Geographical catchments of screening**

The study geographical catchments included two community and one hospital site:

1. Howrah District (three blocks): with a population of 575 961 residing in 240 villages^44^. This site was coordinated by Asha Bhavan Centre (ABC), a non-government community-based disability organisation.
2. Kolkata Municipality (five wards). A migratory community which predominately live in informal settlements (urban slums) with population officially recorded as 352 394^44^. This site was coordinated by the Child In Need Institute (CINI), a non-government community-based child rights organisation.
3. Dr B.C. Roy Post Graduate Institute of Paediatric Sciences is a government hospital providing specialist paediatric services, with a 200-bed Sick Newborn Care Unit. Residents of North 24 Parganas rural district attend this hospital, with recruitment for this site targeting a one hour travel radius surrounding five key communities.

Additional reference

44. Office of the Registrar General & Cenesus Commissioner MoHA, Government of India,. 2011 Census Data 2011 [Available from: https://www.censusindia.gov.in/2011-Common/CensusData2011.html.
